# Combination Therapy for Multidrug-Resistant Klebsiella Pneumoniae Urinary Tract Infection

**DOI:** 10.7759/cureus.1503

**Published:** 2017-07-22

**Authors:** Faizan Yasin, Salman Assad, Abdul Subhan Talpur, Mehr Zahid, Shuja A Malik

**Affiliations:** 1 Neurology, State University of New York at Buffalo; 2 Department of Medicine, Shifa International Hospital, Islamabad, Pakistan; 3 Medicine, Liaquat University of Medical and Health Sciences; 4 Internal Medicine, University of Lahore, Lahore, Pakistan; 5 Internal Medicine, Nawaz Sharif Medical College, University of Gujrat

**Keywords:** multidrug resistant, urinary tract infection, gram-negative bacilli

## Abstract

Hospital-acquired multidrug-resistant (MDR) Klebsiella infection is posing a significant challenge to physicians all around the world. The spread of multiple antibiotic resistance among various members of bacteria continues to be a significant clinical threat. Antibiotic susceptibility testing is the initial step in optimizing the appropriate antibiotic therapy for infections with MDR Klebsiella. We report a case of MDR Klebsiella urinary tract infection (UTI) in a patient following a trimalleolar fracture, which was appropriately treated with a combination of amikacin and meropenem.

## Introduction

Multidrug-resistant (MDR) Klebsiella pneumonia is highly resistant to multiple broad-spectrum antibiotics such as ampicillin and cephalosporins, which were previously helpful in treating these organisms. Hence, its treatment poses a significant challenge to physicians worldwide. The mechanism for its resistance is possibly due to the extended beta-lactamases and carbapenemases produced by these bacteriae [1]. It is mostly hospital-acquired and is seen commonly following invasive surgical procedures. Infections caused by these organisms are not only difficult to treat but also known to cause significant mortality all around the world. They require timely and appropriate antibiotic therapy to improve the patient’s survival [2]. These multidrug-resistant organisms are known to cause various bodily infections such asurinary tract infection (UTI) and pneumonia. Susceptibility testing helps to determine which antibiotics would be appropriate in managing these nosocomial infections. Hence, physicians need to be aware of the possibility of infection with these organisms occurring in a hospital setting and should take all precautionary measures including hand hygiene to help ensure its occurrence is prevented. Antibiotic susceptibility testing will also ensure that extended hospital stays are prevented.

## Case presentation

**Figure 1:**
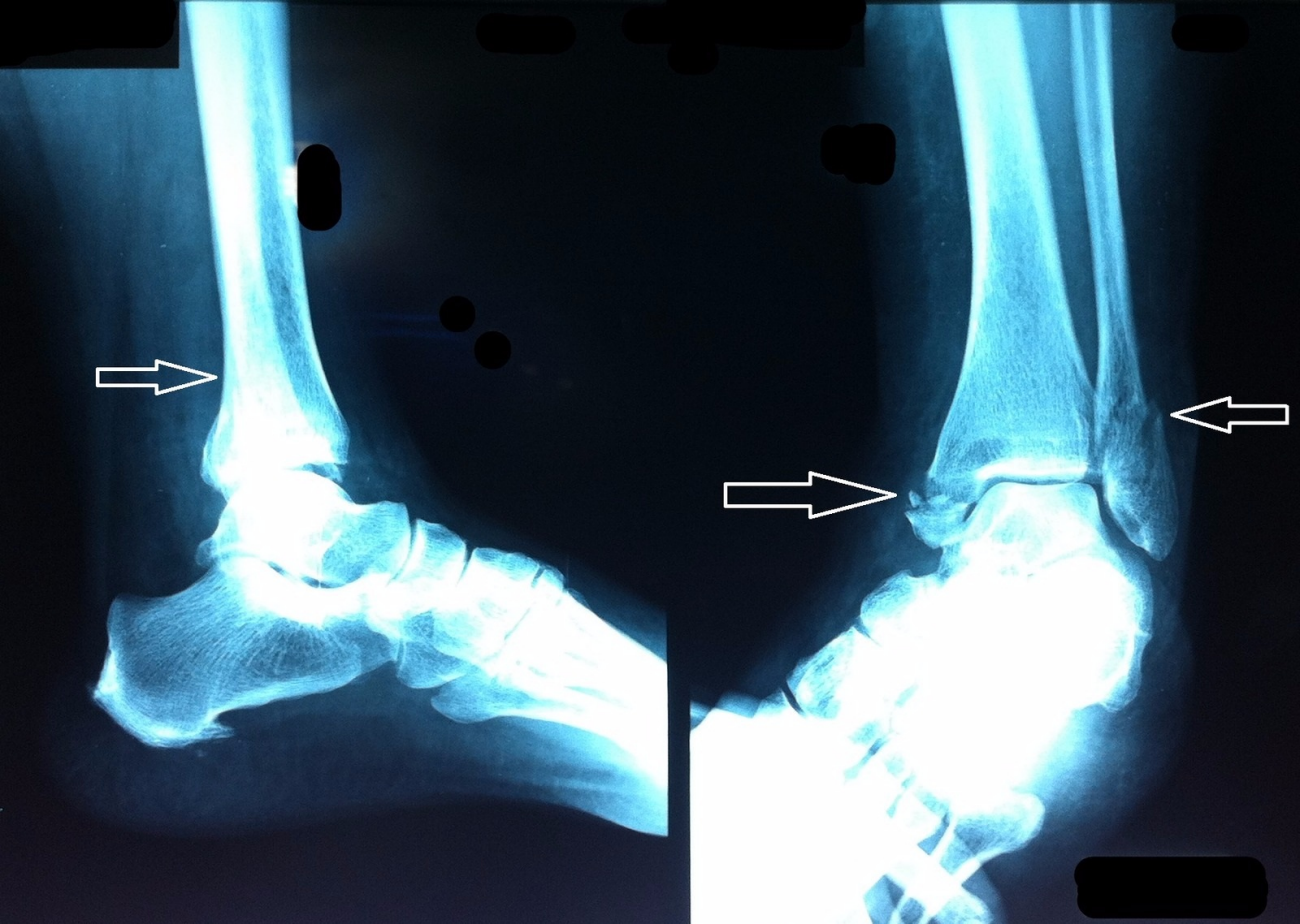
X-ray left foot Left trimalleolar fracture involving the lateral malleolus, the medial malleolus, and the distal posterior aspect of the tibia (white arrows).

A 50-year-old Asian female presented to the orthopedic outpatient department at Ittefaq Hospital (Trust), Lahore after twisting her left foot at home. This traumatic event was followed by immediate swelling, redness, and intense pain in the left ankle region. She was admitted to the orthopedic inpatient ward. An X-ray of the left foot was ordered, which revealed a left trimalleolar fracture - a fracture of the ankle that involves the lateral malleolus, the medial malleolus, and the distal posterior aspect of the tibia (posterior malleolus) (Figure 1). A complete blood count (CBC), blood group testing, electrocardiogram (ECG), liver function tests (LFTs), serum electrolytes, and X-ray of the chest were also ordered. Hepatitis B surface antigen (HBsAg) and anti-hepatitis C virus (Anti-HCV) antibody tests were non-reactive. The CBC differential count revealed increased neutrophils (Table 1) and the erythrocyte sedimentation rate (ESR) was also elevated to 92 mm/hour. Blood urea nitrogen (BUN) and serum creatinine were well above normal values (Table 2). Pain killers were prescribed for pain relief.

**Table 1:** Complete blood picture and differential leukocyte count MCV: mean corpuscular volume, MCH: mean corpuscular hemoglobin, MCHC: mean corpuscular hemoglobin concentration, RDW: red cell distribution width, MPV: mean platelet volume.

Test	Normal Value	Unit	Result
Complete Blood Picture			
White Blood Cells (Count)	4 - 11	10^3/uL	10.80
Red Blood Cells (Count)	4 - 5.2	10^6/uL	4.22
Hemoglobin	11.5 - 16	g/dL	12
Hematocrit	34 - 45	%	35
MCV	79 - 95	fL	82.9
MCH	26 -32	pg	28.4
MCHC	32 -36	g/dL	34.3
RDW	11.5 - 14.5	%	15.3
Platelets	150 - 400	10^3/uL	326
MPV	7.2 - 13	fL	10.1
Differential Count			
Neutrophils	34 - 70	%	77.0
Lymphocytes	19 - 52	%	16.0
Monocytes	2 - 12	%	5.0
Eosinophils	1 - 6	%	2.0

**Table 2:** Laboratory investigations

Test	Normal Value	Unit	Result
Blood Urea Nitrogen (BUN)	10 - 50	mg/dl	54
Serum Creatinine	0.5 - 0.9	mg/dL	1.1
Blood Sugar (Random)	< 140	mg/dL	122
Serum Sodium	135 - 145	mmol/L	136
Serum Potassium	3.3 - 5.1	mmol/L	4.8

Just a month before, the patient was admitted to the intensive care unit (ICU) for acute meningoencephalitis and she was adequately treated for this condition. She had presented with a fever of 100°F, occipital headache and Glasgow Coma Scale (GCS) of 6/15 on admission. Thereafter, she had an episode of vomiting along with neck stiffness. There was no history of focal deficits. She had an altered level of consciousness that progressed to a semi-conscious state and urinary hesitancy. On admission, the patient’s medications included intravenous (IV) ceftriaxone 2 g twice daily, IV acyclovir 250 mg three times daily, omeprazole 40 mg orally twice daily, IV paracetamol 1 g, IV ondansetron 8 mg if there is a need, IV mannitol 150 mg three times daily, IV dexamethasone 10 mg STAT, then 4 g IV hourly, allopurinol 5 mg half dose orally twice daily, levetiracetam 500 mg orally twice daily, nebulized ipratropium bromide eight-hourly, IV vancomycin 1 g twice daily, IV metronidazole 500 mg three times daily, IV dexamethasone 4 mg twice daily, IV midazolam 2 mg STAT, paracetamol plus orphenadrine citrate combination of muscle relaxant orally three times daily, IV diazepam 5 mg STAT and IV normal saline 100 cc/hr. A lumbar puncture was performed successfully after two unsuccessful attempts and it revealed an increased WBC count of 24000/uL, decreased glucose (26 mg/dL) and markedly increased protein (375 mg/dL). These cerebrospinal fluid (CSF) findings were significant for bacterial meningitis. The WBC was markedly elevated to 22,000/uL, C-reactive protein (CRP) was elevated at 74 mg/L and serum albumin level was decreased. On the second day of admission, her urine output had decreased to 15 ml/hr and eventually, she developed urinary stasis that led to the passage of Foley’s catheter. A sample of urine was taken and sent for culture and sensitivity in which no organism was isolated after 48 hours of incubation at 35°C. BUN and serum creatinine values were normal. The patient’s attendant revealed that the three attempts made for lumbar puncture lead to back and leg pain in the aftermath, for which she was prescribed calcium tablets. She was discharged along with the Foley’s catheter with orders of intermittent clamping of the catheter to prevent UTI. She was discharged on esomeprazole 40 mg orally twice daily, paracetamol plus orphenadrine citrate combination of muscle relaxant orally three times daily, IV ceftriaxone 1g orally twice daily, divalproex sodium 500 mg orally three times daily, levetiracetam 500 mg orally twice daily, and dexamethasone 4 mg orally twice daily.

On her recent admission to the orthopedic ward, she presented with a fever of 99°F and was not able to pass any urine on her own, having to rely on Foley’s catheter. The patient revealed that she had not been able to pass urine at all after the fall. She denied urinary frequency, urgency, dysuria, or costovertebral tenderness; however, suprapubic pain was present. Lower abdomen tenderness was noted on examination along with a hard, palpable bladder. The rest of the examination was unremarkable with normal bowel sounds, no guarding or rigidity, normal heart sounds S1 + S2, vesicular breathing, and normal neurologic exam. She did have a history of hypertension for which she had been taking amlodipine. At the time of admission, her blood pressure was 150/100 mm Hg and her BUN and serum creatinine were both raised (Table 3). She had a sensation of bladder fullness; however, she was unable to urinate. The Foley catheter was passed to help her evacuate her bladder. A sample of her urine was sent for culture and sensitivity, which grew isolates of multidrug-resistant Klebsiella. The culture and sensitivity report revealed Klebsiella species, 10^8 CFU/ml, isolated after approximately 24 hours of incubation at 35°C. Antibiotic susceptibilities were determined (Table 4). These were highly resistant isolates of Klebsiella species, being resistant to trimethoprim-sulphamethoxazole (TMP/SMX), ceftriaxone, ampicillin, ceftazidime, cefoperazone, gentamicin, amoxicillin/clavulanic acid, ciprofloxacin with intermediate susceptibility to piperacillin /tazobactam and sulbactam/cefoperazone. They were found susceptible only to amikacin, imipenem, and meropenem. The patient did have a history of hesitancy in her previous admission in the ICU for acute meningitis, a month ago; however, the urine culture revealed no organism at the time.

**Table 3:** Laboratory findings

Test	Normal Value	Unit	Result
Blood Urea Nitrogen (BUN)	10 - 50	mg/dL	34
Serum Creatinine	0.5 - 0.9	mg/dL	0.8

**Table 4:** Antibiotic susceptibilities R: Resistant, S: Sensiitive.

Antibiotic(s)	Susceptibility
Amikacin	S
Imipenem	S
Meropenem	S
TMP/SMX	R
Ceftriaxone	R
Ampicillin	R
Ceftazadime	R
Cefoperazone	R
Gentamicin	R
Amoxicillin/Clavulanic acid	R
Ciprofloxacin	R
Piperacillin/Tazobactem	S
Sulbactam/Cefoperazone	S

**Figure 2:**
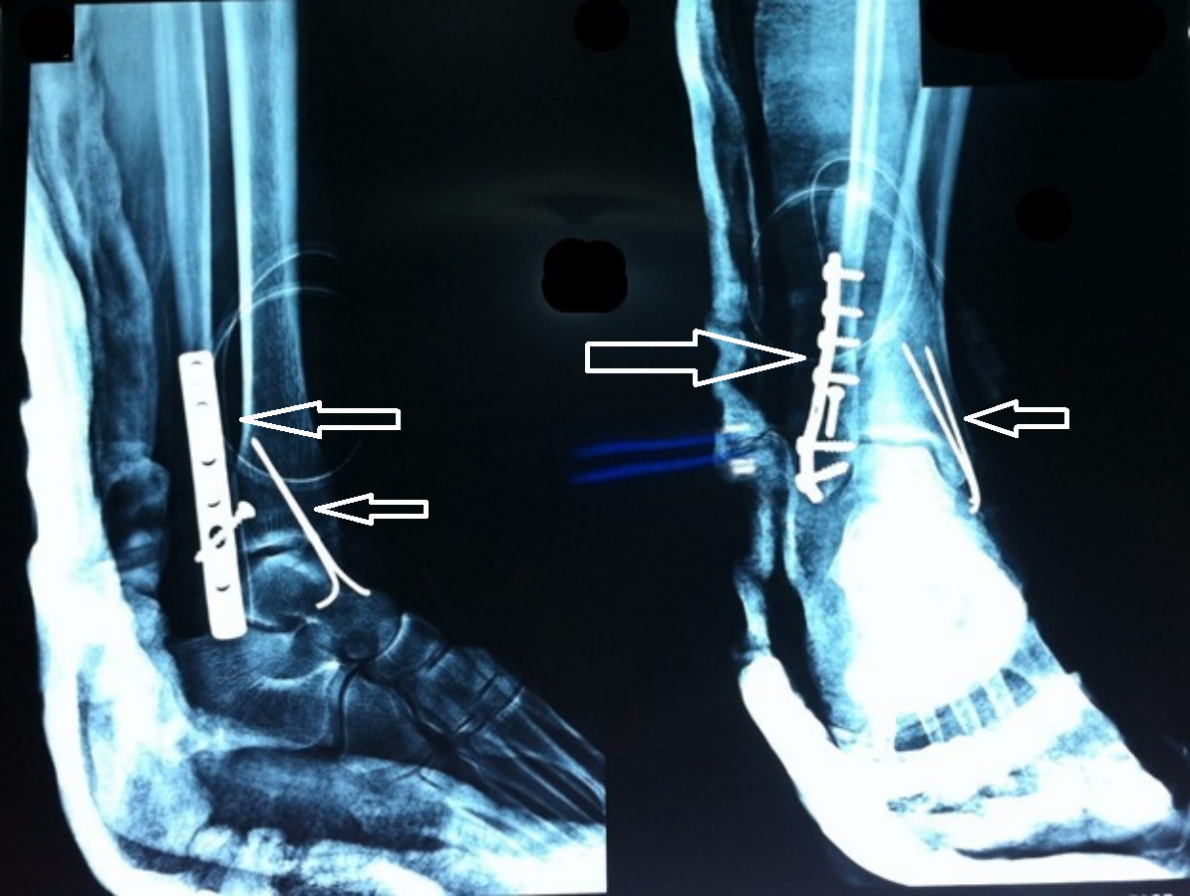
X-ray left foot After open reduction and internal fixation (white arrows).

She underwent open reduction and internal fixation of the left ankle by the orthopedic surgeon (Figure 2). Postoperatively, the patient was vitally stable. Physiotherapy was advised for the patient and the patient was mobilized following the operation. There was no complaint of fever; however, she did complain of generalized weakness which lead to a neurology consultation. Neurobion injection that contains B vitamins, B1, B6, and B12 was delivered to the patient to boost her energy levels. On admission, the patient’s medications included tramadol HCL plus paracetamol combination orally twice daily; IV pantoprazole 40 mg twice daily; IV amikacin 500 mg twice daily; sulfolax (laxative drops), intramuscular vitamin B12, intramuscular vitamin D3 once weekly, calcium, vitamin C, vitamin B6, oxcarbazepine 300 mg half dose orally twice daily, phenytoin 250 mg orally three times daily, and IV meropenem 500 mg thrice daily. The anti-epileptics were continued from her previous admission in the ICU for acute meningitis when she had a few episodes of seizures. Blood urea nitrogen and serum creatinine were ordered on third and sixth postoperative day, which came out normal on both occasions (Table 5). Hemoglobin was slightly low. The Foley’s catheter was removed and she was finally discharged on the tenth postoperative day in stable condition.

**Table 5:** Following trend of BUN and serum creatinine

Test	Normal Value	Unit	Result
Blood Urea	10 - 50	mg/dL	27
Serum Creatinine	0.5 - 0.9	mg/dL	0.8

## Discussion

Multidrug-resistant gram negative organisms have been known to cause significant morbidity and mortality worldwide. Susceptibility testing has proven to be an effective way for optimizing antibiotic therapy for treating such infections. In our case presentation, we have described the management strategy for MDR Klebsiella pneumonia in a patient following a hospital stay due to lower limb fracture. This case presentation will also serve as a potential treatment strategy for future similar cases. Our patient experienced urinary hesitancy as the only complaint about her UTI. Urinary hesitancy is usually noticed when the person is not able to urinate which results in urinary retention. This often leads to discomfort in the suprapubic region and swelling of the bladder. It can also result as a side effect of surgery as might be the case in our patient. Besides, it can also result from stress and anxiety, especially in women, as might be the case here due to the foot fracture causing significant anxiety in our patient. Acute retention of urine can cause anuria. Infection with multidrug-resistant pathogens is more likely to occur in UTI complicated by an obstruction or any factor that causes urinary stasis, for example, urolithiasis, malignancy or neurogenic bladder. Use of inhaled anticholinergic drugs also increases the risk of acute urinary retention. The risk of acute urinary retention (AUR) is highest in recent starters and in patients receiving their anticholinergic drugs via nebulizer [3]. Our patient also received nebulized ipratropium bromide eight-hourly during her previous admission for meningoencephalitis. Additionally, the patient was previously exposed to broad spectrum antibiotics like ceftriaxone in the last couple of months. We must conserve the efficacy of existing antibacterial drugs as much as possible to eliminate this emerging health problem [4].

## Conclusions

Klebsiella pneumonia isolates resistant to most of the commonly used antibiotics have been recognized as emerging infectious organisms of clinical significance. The worldwide spread of this organism is proving to be quite a challenge for physicians who are left with limited therapeutic resources when dealing with such cases. We have described the management strategy for an MDR Klebsiella UTI and potential strategies to manage these types of infections in future patients. Treatment strategies evaluated to treat MDR Klebsiella species in UTI include combination therapy with amikacin and meropenem. It is also critical to have protocols in place for effective infection control efforts to limit the spread of these pathogens.
